# Pulp Sensitivity Testing in Multiple Sclerosis: Disease Duration and Sensory/Motor Associations—A Cross-Sectional Study

**DOI:** 10.1155/2024/6662518

**Published:** 2024-09-11

**Authors:** Fatemeh Owlia, Fereshteh Noori, Marzieh Abutorabi Zarchi, Maryam Kazemipoor

**Affiliations:** ^1^ Department of Oral and Maxillofacial Medicine School of Dentistry Shahid Sadoughi University of Medical Sciences and Health Services, Yazd, Iran; ^2^ Department of Neurology School of Medicine Shahid Sadoughi University of Medical Sciences and Health Services, Yazd, Iran; ^3^ Department of Endodontics School of Dentistry Shahid Sadoughi University of Medical Sciences, Yazd, Iran

**Keywords:** dental pulp, diagnosis, electric pulp test, multiple sclerosis, thermal pulp test

## Abstract

**Introduction:** This study explores a relatively unexplored aspect of multiple sclerosis (MS) by examining the sensitivity threshold of dental pulp as a potential indicator of neuropathy in MS patients. Building upon earlier research that focused on assessing the response to electrical pulp testing in MS patients who did not have a history of trigeminal neuralgia, this survey is aimed at delving into the relationship between MS duration and the threshold for stimulation in response to pulp sensitivity tests.

**Materials and Methods:** This study encompassed a total of 124 maxillary central incisors from patients diagnosed with relapsing-remitting multiple sclerosis (RRMS). The participants were uniform in terms of age, falling within the 18–50 years range, and all had RRMS with no history of trigeminal neuralgia. The electric pulp sensitivity test was conducted on all samples, and the results of the electric pulp testing (EPT) were recorded according to the grade of the pulp tester that elicited a response. The threshold was considered reached when the patient first experienced a burning sensation after EPT application and the use of 1,1,1,2-tetrafluoroethane spray. Data analysis employed paired *t*-tests, Fisher's exact test, and Spearman correlation, with a significance level set at *p* < 0.05.

**Results:** Based on the study's findings, the average response value to EPT was 2.69 ± 1.17, while the response time to the cold test was 2.61 ± 1.03 s. There was no statistically significant difference in the response to the cold test based on age (*p* = 0.45). However, it was observed that the mean response time to the cold test was significantly longer among male participants (*p* = 0.001). No significant differences were identified in the pulpal response to EPT or the cold test between patients with and without sensory-motor involvement (*p* > 0.05). Furthermore, Spearman's analysis revealed a noteworthy positive correlation between the electrical pulp threshold and the time taken to respond to the cold test (*p* = 0.025, *r* = 0.2).

**Conclusions:** The utilization of the pulpal sensitivity test in MS patients holds promise for practical clinical use. Notably, individuals with a more extended duration of the disease exhibited a notably elevated threshold for both the EPT and the cold test conducted on their maxillary central incisors.

## 1. Introduction

Multiple sclerosis (MS) stands as the most prevalent chronic inflammatory disease affecting the central nervous system (CNS) [1]. Typically, it strikes individuals in the age bracket of 20–40 years [2, 3], with a notable predominance in women, with MS being three times more common in females than males [4]. It is characterized by inflammation and demyelination of nerve fibers. This complex condition presents with a range of symptoms, including sensory disturbances, motor impairments, cognitive difficulties, and fatigue [5].

Regarding prevalence, Iran is classified as a low-risk region for MS, with a prevalence rate of less than 5 per 100,000 [2]. Surveys, such as the Atlas survey, have revealed significant variation in the age of diagnosis among different countries, but when comparing mean ages across various time points, no discernible trend towards earlier diagnosis on a global scale is evident [6]. Although there has been progress in understanding the pathophysiology of MS, the exact mechanisms causing specific symptoms, particularly sensory impairments, are still not fully understood [7]. Sensory dysfunction is a common and distressing problem in MS that greatly affects the quality of life for patients. These issues can range from mild numbness and tingling to severe pain and loss of feeling [8]. Despite the significant impact of sensory symptoms, there has been limited research in this area, particularly when it comes to the involvement of the CNS. Peripheral neuropathies, which may contribute to sensory impairments in MS, have not been extensively studied.

Oral manifestations of MS can manifest as facial paralysis or spasms, trigeminal neuralgia, dysphagia, temporomandibular joint (TMJ) complications, and xerostomia, all of which can adversely affect an individual's quality of life [9]. Although some studies have reported a higher prevalence of dental caries among MS patients, the elevated rate of DMFT (decayed, missing, filled teeth) in these individuals underscores the substantial impact that MS has had on their dental health and overall quality of life over the years [3].

It is essential to understand the connection between peripheral sensory function and MS for multiple reasons. Firstly, this understanding can shed light on the initial phases of disease progression, as damage to peripheral nerves may occur before or alongside CNS harm [10]. Secondly, by assessing peripheral sensory function, we may discover potential biomarkers for monitoring disease and gauging treatment response. With early indicators of disease progression, clinicians can intervene more effectively to slow or stop its advancement [11].

Sensitivity pulp tests (thermal and electrical pulp test [EPT]) are still widely used by dentists to determine the state of pulp health in permanent teeth [12]. These tests have gained popularity nowadays to clarify some wounder results of clinical studies [13]. The cold test is a specific test to evaluate pulp sensitivity. It is one of the best diagnostic methods used due to its high accuracy and repeatability [14]. Cold testing is thought to stimulate Type A-delta fibers in the dental pulp, which elicit a sharp, localized pain. This test does not affect the C-fibers of pulp tissue, except in teeth with irreversible pulpitis [14].

The EPT mechanism operates based on the concept that electrical stimuli induce an ionic alteration across neural membranes. This phenomenon results in rapid signal propagation at the nodes of Ranvier, particularly in myelinated nerves [15]. In neurodegenerative disorders such as MS, nerve function may become compromised, potentially disrupting sensory impulse transmission. These alterations can influence the outcomes of EPT [16]. Ensuring an accurate and prompt diagnosis of MS, along with the recognition of its associated oral complications, can help prevent unforeseeable irreversible consequences [17]. Although great effort has been made on the sensory nerve of several diseases such as celiac, diabetes, and MS since many years ago [18, 19], to the best of our knowledge, evaluating tooth somatosensory evoked potentials in MS has been neglected. The previous study reported that the response to EPT of MS patients was significantly decreased [20]. Given the existing gap in research on this subject, this survey serves as an initial step toward the design of future experiments. It builds upon previous investigations into the assessment of responses to electrical pulp testing in MS patients who do not have a history of trigeminal neuralgia [20]. The potential alterations identified in this study hold promise for facilitating the timely diagnosis of sensory changes associated with MS. Furthermore, this survey is aimed at elucidating the stimulation threshold in response to pulp sensitivity tests, drawing insights from the duration of MS.

## 2. Methods

### 2.1. Sample Size Calculation and Sampling Method

According to Owlia et al.'s study [20] which had an SD = 0.567, the researchers determined the standard deviation of the electrical stimulation threshold score. With 95% confidence (*α* = 5%) and precision (*d* = 0.10), a sample size of 124 people was obtained.

Regarding the study's design, patients were referred to a specialized registered center for MS patients located in Yazd, Iran, where they were under the care of an experienced neurologist. Initially, a total of 320 patients diagnosed with relapsing-remitting MS were considered. These patients were entered using convenience sampling. After applying the inclusion criteria, 196 of them did not meet the requirements. Eventually, 124 eligible participants, consisting of 45 males and 79 females, were selected for the research. The research started in April 2021 and ended in June 2021.

### 2.2. Inclusion and Exclusion Criteria

These inclusion criteria stipulated that participants must have relapsing-remitting MS, have an age range of 18–50 years, be clinically stable for at least 6 months since their last clinical attack, exhibit normal trigeminal nerve examination results, and have no sensory or motor symptoms related to the trigeminal nerve. All clinical examinations and paraclinic tests were overseen by an expert neurologist.

Patients who had a history of recent use of anti-inflammatory drugs or medications with neuropathic side effects in the past 3 months, a history of drug abuse, and the existence of other systemic diseases with neuropathy symptoms, such as diabetes or nutritional/metabolic disorders, would be excluded from the study.

It is worth noting that despite receiving various disease-modifying treatments (DMTs), all patients had been clinically stable (without any relapse symptoms) for at least the past year, as confirmed by the neurologist.

Additionally, intact maxillary central teeth with no restorations, attrition, caries, history of trauma, or orthodontic treatment were chosen for the survey. Teeth with discoloration or signs of periodontal disease were excluded from the study.

### 2.3. Ethical Consideration

The study was carried out by the Declaration of Helsinki guidance of the World Medical Association and Good Clinical Practice recommendations. At first, the purpose and the method of the survey were fully teased out to the participants, and they were also informed that they could leave the study at any stage of the work. They entered the study willingly and were able to work with the examiner. The information was provided to participants, and any potential risks or benefits were described. The volunteer patients signed the written informed consent before entering the study. All participants filled out the HAD (Hospital Anxiety and Depression) scale questionnaire to confirm a matched condition of anxiety. The Committee of Ethics for Human Research approved the study at Shahid Sadoughi University of Medical Sciences, Yazd, central Iran, and received the code of ethics: IR.SSU.REC. 1399.219.

### 2.4. Measurements

Before the study's commencement, a comprehensive history of symptoms and physical examinations was conducted. Information collected included age, gender, disease duration, and self-reported presence or absence of clinical sensory or motor symptoms in the limbs or face.

All the pulp test procedures were by the last year's dental student trained for pulp testing.

**Figure 1:**
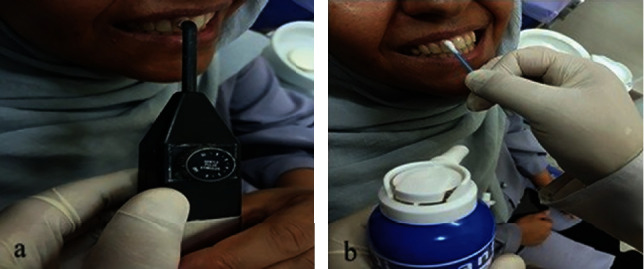
Pulpal sensitivity tests. (a) Electric pulp testing. (b) Cold test.

For the EPT, the procedure involved isolating, cleaning, and drying the teeth using sterilized cotton rolls. A lip clip was attached to the corner of the lip, and the probe tip of the electric pulp tester (PARKELL, Edgewood, NY-11717T, Battery 9 V, made in the United States) was coated with prophylactic (Golchai, Iran) before being applied to the one-third incisal edge of the buccal surface of the tooth crown (Figure 1). The probe tips which have been applied are sterilized before each test. The device was activated, and the voltage gradually increased. This process continued until the patient reported feeling a tingling or burning pain for the first time. At this point, the device was removed from the tooth, and the number displayed on the pulp tester was recorded as the EPT threshold.

For the cold test, 1,1,1,2-tetrafluoroethane spray (Denronic brand, made in Germany) was used (Figure 1). Complete isolation was achieved using a cotton roll, and the cotton head of the swabs was sprayed from 30 cm with one puff. The swab was then placed on the one-third incisal edge of the labial surface of the central maxillary tooth. Rubbing the swab continued until the patient experienced pain. The sterilized cotton ball was pressed against the tooth for approximately 3 s, and the stimulus position was maintained to determine either an immediate tooth response or a response within 10 s. Both test procedures were carried out under strict infection control and sterile conditions.

### 2.5. Statistical Analysis

The data for this study were gathered and organized into categories, which included age, gender, sensory-motor symptoms, duration of MS, and responses related to the teeth. Data falling outside the normal range was excluded. The comparison of numbers obtained from the EPT and cold tests was analyzed using SPSS17 (SPSS17; Chicago, IL, United States). Results were presented as means and standard deviations. The primary statistical tests used, including paired *t*-test, Fisher's exact tests, and Spearman correlation, were employed to assess the significance of differences in variables among participants and to evaluate the relationship between the EPT threshold and the time taken to respond to the cold test (*p* < 0.05).

## 3. Results

A total number of 124 patients were enrolled in the work, 79 (63.7%) patients were female, and 45 (36.3%) patients were male (Table 1). The mean ± SD of age in patients was 38.15 ± 7.21 years with a range of 18–50 years. Noting the findings of the study, 108 (87.1%) patients had clinical, sensory, or motor symptoms (Table 1).

The mean ± SD duration of MS was 8.16 ± 4.88 years. Overall, the results presented that the mean threshold of EPT was higher in the older group, but no statistically significant difference was detected between them (*p* = 0.06) (Table 2).

**Figure 2:**
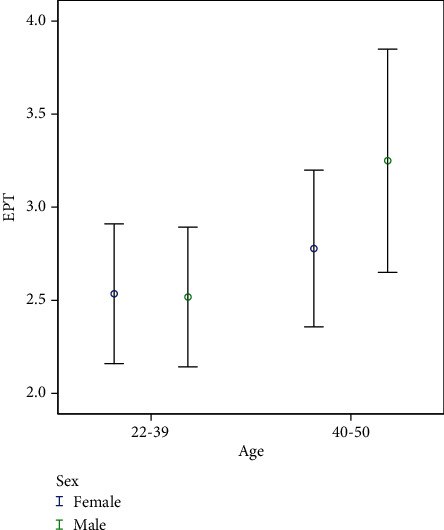
Relationship between ages with EPT.

As can be seen from Table 3, the response rate to the cold test and the duration time of responding to this test are displayed. No meaningful difference was observed between the responses to the cold test based on age (*p* = 0.45). Nonetheless, the duration time in response to the cold test was significant (*p* = 0.01) (Figure 2).

Results of the paired *t*-test delineated that despite a light mounting score of EPT in the older group, it had no significant difference (*p* = 0.06).

**Figure 3:**
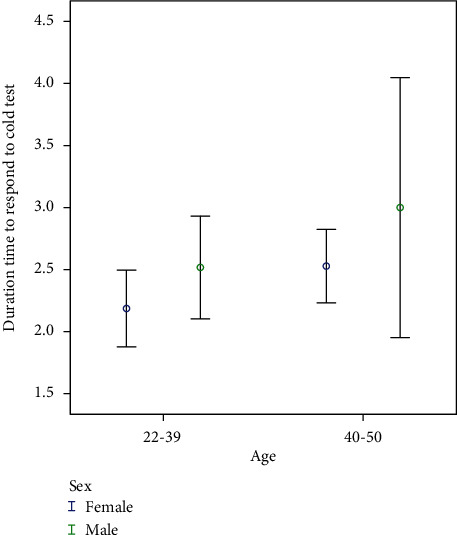
Relationship between age groups with duration of delay in response to cold test.

A slightly higher rate of EPT responses was observed in men, but this difference was not statistically significant (*p* = 0.55). Conversely, the relative frequency of positive responses to the cold test was higher in women, although the Fisher exact test did not reveal a significant difference (*p* = 0.25). Notably, there was a significant difference in the mean duration of response delay to the cold test between the two genders (*p* = 0.001). This indicates that, on average, men took significantly longer to respond to the cold test (as illustrated in Figure 3).

Based on the mean EPT threshold, an insignificant difference was discerned between patients with and without sensory or motor symptoms. The relative frequency of positive response to the cold test and duration time to the cold test, among individuals with sensory or motor symptoms and in asymptomatic individuals, was insignificant.

**Figure 4:**
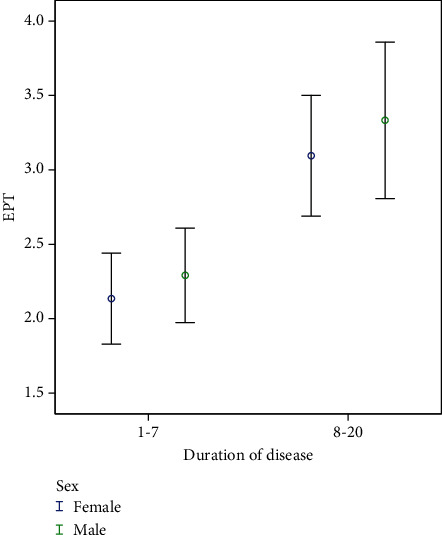
Relationship between duration of disease and EPT.

**Figure 5:**
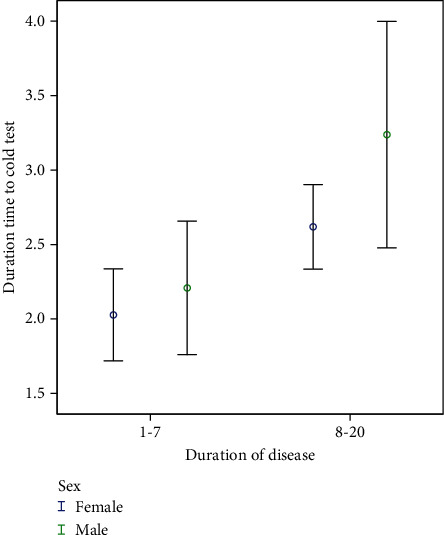
Relationship between duration of infection and duration of delay in response to cold test.

Based on our findings, the mean threshold of EPT was higher in patients with a longer duration of MS with a meaningful difference (*p* = 0.001). Regarding the duration of time to respond to the cold test, the difference was significant, too. This rate was longer in patients with a longer duration of MS (*p* = 0.001). Although a slightly higher rate of positive response to cold test in patients with a longer duration of MS was recognized slightly, it was insignificant (*p* = 0.71) (Table 3) (Figures 4 and 5).

Intriguingly, that response to EPT was significantly correlated with the positive correlation evident for the duration time to respond to the cold test (*p* = 0.025) (Spearman correlation coefficient 0.2).

## 4. Discussion

MS stands out as one of the most prevalent inflammatory demyelinating diseases, leading to degenerative nerve changes [21]. This disease can manifest in both visible and hidden ways, depending on the nerves affected within the brain or spinal cord [22]. Among these nerves, the trigeminal nerve is frequently implicated in MS cases [23]. However, there has been limited exploration of how this disease, its treatment, and resulting sensory or motor symptoms impact patients' responses to pulp sensitivity tests in previous studies [20]. Previous research has indicated that MS patients exhibit significantly reduced responses to EPT compared to healthy individuals [24].

Pulp sensitivity tests encompass both thermal and electrical assessments. Among these, the cold test holds high diagnostic sensitivity, with the electrical test ranking second in terms of diagnostic value [14]. The mechanism underlying the stimulation of pulpal nerves during the cold test involves the activation of A-delta nerve fibers within the dentinal tubules through a hydrodynamic process. In contrast, the EPT directly triggers A-delta fibers within the pulp complex. The combined results obtained from pulp sensitivity tests offer valuable insights into the condition of the dental pulp, aiding clinicians in making informed judgments for more effective treatment strategies [20].

MS, as a demyelinating disease, can affect the body's sensory nerves, including the trigeminal nerve. According to some studies, nerve involvement can vary depending on the duration of the disease [25]. In other words, the effect of the duration of MS on the course of the disease and the occurrence of its symptoms have been contradictory [25]. This survey expanded its scope by including the cold test in addition to EPT and incorporated a larger sample size, along with more detailed inquiries related to the duration of the disease in MS patients. The primary goal of this study was to assess how responses to pulp sensitivity tests were influenced by various variables in MS patients.

In MS clinical trials, the annualized relapse rate is commonly used as a primary outcome measure, and it has been shown to decrease with treatment but does not reach zero [26]. These relapses can have an impact on disability, particularly in the short-term period [9]. As the duration of the disease in MS patients increases, there is a corresponding rise in disease progression and neurodegenerative changes within the CNS [27]. This prolonged duration of the disease can result in more sensory and motor symptoms and potentially greater disruption of the trigeminal nerve in the brainstem. This study is aimed at investigating the subclinical dysfunction of this nerve by excluding patients who already exhibit clinical disorders of the trigeminal nerve.

The maxillary central tooth was opted as the suitable location for exploring the nerve stimulation threshold of the trigeminal nerve. Due to the more accessible position, easier isolation, less probability of caries, point connection to adjacent teeth, and lower threshold to EPT, the central maxillary incisor was selected [28]. Because of the accumulation of neural network, enamel thickness, and the direct path of dentinal tubules in one-third incisal edge of the labial surface of the central tooth, this area opted for EPT evaluation as this requires the minimum voltage to stimulate the tooth [29, 30]. An experiment asserted that trigeminal sensory-motor evoked potentials may indicate latent MS lesions and may be the early sign of the presence of pathological changes in MS patients [31]. Plenty of studies measuring the effect of MS duration on electrophysiological changes have yielded contradictory results [32, 33]. In Owlia et al.'s research, it was noted that while there was a positive association between the duration of MS and tooth responses to EPT, this relationship did not reach statistical significance [20]. In another study, it was demonstrated that responses to electrical tests may serve as a more sensitive indicator than other tests in detecting the demyelination process of nerve fibers [34]. Dysfunction of the trigeminal nerve can disrupt the response of pulp sensitivity tests because the sensory terminals of the stimulated tooth are connected to the terminal branches of the trigeminal nerve [12].

Upon closely examining the results of this survey, a noteworthy observation is that patients with a longer history of MS displayed delayed responses to both cold and electrical tests. Additionally, these delayed responses were more pronounced in men across both age groups. This suggests that male patients may exhibit greater sensitivity to changes induced by the duration of MS when compared to women of the same age group, potentially hinting at varying rates of damage that warrant exploration in future studies. It is worth noting that in this survey, there was no significant gender-based difference in response to the cold test and EPT, a finding consistent with previous research [35]. However, there was a significant gender difference in the response delay to the cold test, suggesting that men were less sensitive to cold stimulus compared to women.

While some prior studies have assessed the cold test solely in terms of a positive or negative response, this survey, in line with certain other studies, evaluated the duration of time taken to respond to the cold test [36]. Interestingly, the response time to the cold test in men exhibited a wider range due to varying dentin thickness [37].

Furthermore, the most favorable responses to pulpal sensitivity tests were observed in patients under the age of 50 years, aligning with the age range of 18–50 years for participants in this study. This is consistent with previous research that also found no statistically significant differences in dental pulp sensitivity tests between two age groups [35]. Our findings further support this trend. Additionally, Farac et al. [36] noted that older individuals had longer response times to the cold test, which was similarly observed in our experiments, where the older age group exhibited a significantly longer response delay to the cold test.

In this study, any evidence of sensory or motor disturbance in the face or limbs, either recorded in the patient's file, based on recent regular tests, or any symptoms expressed based on clinical examination was considered positive. Findings revealed that there was no significant difference in the tooth response to the cold test, EPT, and duration time in response to the cold test between patients with and without sensory or motor symptoms. Regarding the kind of literature, peripheral nerve stimulation can occur in patients suffering from MS even in the absence of clinical sensory or motor symptoms [20]. Trigeminal nerve dysfunction can cause a disturbance in the response of the pulp sensitivity. Trigeminal nerve dysfunction can cause a disturbance in the response of sensory terminal within the pulp complex to pulpal sensitivity tests [38]. The purpose of this study is to prove the subclinical disorder of this nerve by screening the patients who had clinical disorders in the trigeminal nerve. Although individuals with sensory or motor symptoms responded for a longer time to EPT and cold tests, no statistically significant difference was detected.

The progression of MS shares similarities with chronic conditions like diabetes, and it is worth noting that the true duration of the disease may be underestimated [39]. Typically, the onset of the disease is considered as the first time a patient perceives any signs or symptoms. The impact of disease duration on its progression has been a subject of debate in various studies with contradictory findings [40, 41].

Interestingly, in our study, we found no significant relationship between the response to the cold test and the duration of MS. However, individuals with a longer duration of MS exhibited a significant increase in the time it took to respond to the cold test. This finding aligns closely with the results reported by Kale et al. [32]. Furthermore, our results support the notion put forth by Owlia et al. that with a larger population size, a significant positive relationship between disease duration and the response to EPT might become apparent [20]. This could be attributed to the fact that the cold test stimulates nerves through a hydrodynamic process that is not influenced by the duration of MS. In contrast, EPT directly stimulates nerves, and as the disease progresses and demyelination occurs, nerve stimulation may be altered. Research has indicated that, compared to MRI imaging, assessing evoked potentials from tongue somatosensory nerves is a suitable method for detecting deterioration and early damage to the afferent trigeminal nerve in MS patients [42].

When evaluating patients without trigeminal neuralgia, we found significant differences in the response time to the cold test based on age, gender, and disease duration. Moreover, the threshold of EPT was significantly higher in patients with a longer history of the disease compared to other groups. In contrast, the response to the cold tests showed no significant differences concerning age, gender, the presence or absence of sensory or motor symptoms, or disease duration. However, it is important to exercise caution when interpreting these findings.

To mitigate probable biases, the following points were considered:

– To avoid measurement bias such as interrater reliability between examiners in this study one person (the last year dental student trained for pulp testing did the procedure).
– To avoid selection bias, the study design was well-structured, and specific inclusion or exclusion criteria for patient selection were defined in detail.

Several limitations may have influenced the results of this study. These include a relatively small sample size and various methodological shortcomings. Constraints such as limited operating hours of the MS clinic, reliance on archived patient documents, omission of MRI reports in the patient documents, and challenges posed by the patients' reduced cooperation during the COVID-19 pandemic were notable deficiencies in this survey.

To address these limitations, future research could benefit from multicenter studies and a more in-depth exploration of specific symptom groups. This approach would help mitigate confounding factors such as age, dentin thickness, and the potential influence of female hormones on pulpal sensitivity responses.

## 5. Conclusion

Given the considerable prevalence of trigeminal nerve involvement in MS patients, alterations in tooth stimulation thresholds, acting as sensory nerve terminals, may serve as indicators of sensory-motor changes in the subclinical stage among suspected patients. EPT and the cold test could be suggested as supplementary assessments to aid clinicians in their evaluations. Notably, individuals with an extended disease duration exhibited significantly higher thresholds for both EPT and cold tests in their teeth. It is important to note that the conclusions drawn from this investigation are subject to certain limitations.

## Figures and Tables

**Table 1:** Frequency of gender and sensory-motor symptoms in patients.

**Number (percent)**	**Scale**	**Variables**
45 (36.29%)	Men	Gender
79 (63.7%)	Women	
30 (27.8%)	Sensory	Sensory-motor symptoms
42 (38.9%)	Motor	
36 (29.0%)	Sensory motor	
16 (12.9%)	None	

**Table 2:** Pulp response to cold and EPT tests.

**M** **e** **a** **n** ± **SD**/**N (%)**	**Scale**	**Variables**
117 (94.4%)	Positive	Response to cold test
7 (5.6%)	Negative	
1.03 ± 2.61	Second	Duration of response to cold test
1.17 ± 2.69	Score	Electrical stimulation threshold of the pulp

**Table 3:** Frequency, mean, and standard deviation of the studied variables in patients.

**Electrical stimulation threshold of the pulp (mean ± SD)**	**Response to cold test**		**Duration of response to cold test (mean ± SD)**
	**Positive** **N** ** (%)**	**Negative** **N** ** (%)**	
1.12 ± 2.52	69 (95.8%)	3 (4.2%)	0.94 ± 2.42
1.21 ± 2.92	48 (92.3%)	4 (7.7%)	1.09 ± 2.89
1.17 ± 2.69	117 (94.4%)	7 (5.6%)	1.03 ± 2.61
0.06^a^	0.45^b^		0.01^a^
1.08 ± 2.77	41 (91.1%)	4 (8.9%)	1.24 ± 2.95
1.23 ± 2.64	76 (96.2%)	3 (3.8%)	0.85 ± 2.43
1.17 ± 2.69	117 (94.4%)	7 (5.6%)	1.03 ± 2.61
0.55^a^	0.25^b^		0.001^a^
1.15 ± 2.73	102 (94.4%)	6 (5.6%)	1.01 ± 2.65
1.31 ± 2.43	15 (93.8%)	1 (6.3%)	1.11 ± 2.33
1.17 ± 2.69	117 (94.4%)	7 (5.6%)	1.03 ± 2.61
0.35^a^	1.00^b^		0.25^a^
0.85 ± 2.19	57 (93.4%)	4 (6.6%)	0.82 ± 2.24
1.25 ± 3.17	60 (95.2%)	3 (4.8%)	1.08 ± 2.96
1.17 ± 2.69	117 (94.4%)	7 (5.6%)	1.03 ± 2.61
0.001^a^	0.71^b^		0.001^a^

^a^ *t*-test.

^b^Fisher exact test.

## Data Availability

All data analyzed during this study is included in this published article. If any, additional data/files may be obtained from the corresponding author on reasonable request.
